# League of Mercy Garden Party

**Published:** 1908-07-04

**Authors:** 


### LEAGUE OF MERCY GARDEN PARTY.

In accordance with their custom, the Prince and Princess of Wales received some of the members of the League of Mercy at Marlborough House on June 24. The weather was ideal for an outdoor function, and the lawns presented a bright appearance when the Prince and Princess appeared among their guests. Their Royal Highnesses were accompanied by Prince Henry and Princess Mary of Wales, and by Princess Louise of Schleswig-Holstein and Princess Alexander of Teck. A number of ladies and gentlemen then received the badge of the Order of Mercy from the hands of the Prince. Refreshments were then served. Previously about 140 of the Presidents and Lady Presidents of the League had assembled in the saloon of Marlborough House, and the report of the year's working had been read by Mr. J. Harrison, M.V.O., one of the hon. secretaries. This showed that during the last year the League had given £18,000 to the King's Hospital Fund. In replying, the Prince of Wales mentioned that since its incorporation in 1899 the League had contributed nearly £100,000 to the hospitals of London and the home counties. Of recent years the League has collected subscriptions from more remote districts than at first, and has in return made grants to the local hospitals of the different neighbourhoods. These grants, which amounted only to £15 15s. in 1901, last year reached the sum of £1,006, and this year an even greater sum will be granted to these provincial hospitals. The Prince concluded by thanking the ladies and gentlemen who got up the concert at the Albert Hall to which workers for the League were invited, and expressed his satisfaction that, while several large donations had been received the small subscriptions of a shilling and upwards which the League was formed to draw into the coffers of the hospitals are being well maintained.

